# Comparing Efficiency of Lysis Buffer Solutions and Sample Preparation Methods for Liquid Chromatography–Mass Spectrometry Analysis of Human Cells and Plasma

**DOI:** 10.3390/molecules27113390

**Published:** 2022-05-25

**Authors:** Lasse Neset, Gracious Takayidza, Frode S. Berven, Maria Hernandez-Valladares

**Affiliations:** 1 The Department of Biomedicine, University of Bergen, Jonas Lies vei 91, 5009 Bergen, Norway; lasse.neset@student.uib.no (L.N.); gracious.takayidza@student.uib.no (G.T.); frode.berven@uib.no (F.S.B.); 2 Department of Clinical Science, University of Bergen, Jonas Lies vei 87, 5021 Bergen, Norway; 3 Department of Physical Chemistry, University of Granada, Campus Fuentenueva s/n, 18071 Granada, Spain

**Keywords:** sodium dodecyl sulfate, guanidinium hydrochloride, liquid chromatography–mass spectrometry, proteomics, single-pot, solid-phase-enhanced sample preparation, in-solution digestion, plasma, cells, depletion

## Abstract

The use of a proper sample processing methodology for maximum proteome coverage and high-quality quantitative data is an important choice to make before initiating a liquid chromatography–mass spectrometry (LC–MS)-based proteomics study. Popular sample processing workflows for proteomics involve in-solution proteome digestion and single-pot, solid-phase-enhanced sample preparation (SP3). We tested them on both HeLa cells and human plasma samples, using lysis buffers containing SDS, or guanidinium hydrochloride. We also studied the effect of using commercially available depletion mini spin columns before SP3, to increase proteome coverage in human plasma samples. Our results show that the SP3 protocol, using either buffer, achieves the highest number of quantified proteins in both the HeLa cells and plasma samples. Moreover, the use of depletion mini spin columns before SP3 results in a two-fold increase of quantified plasma proteins. With additional fractionation, we quantified nearly 1400 proteins, and examined lower-abundance proteins involved in neurodegenerative pathways and mitochondrial metabolism. Therefore, we recommend the use of the SP3 methodology for biological sample processing, including those after depletion of high-abundance plasma proteins.

## 1. Introduction

In recent years, advances in mass spectrometry (MS)-based proteomics, and its high sensitivity and selectivity in the identification and isolation of peptides, propelled it to be the predominant technology in the analysis of proteomes [1]. Bottom-up shotgun analysis is the most-used MS technology, whereby proteins are cleaved into small peptides using sequence-specific enzymes, the mixture is then fractionated, and further identification is conducted using coupled liquid chromatography–MS (LC–MS) [2]. LC–MS proteomics enable the investigation of biological processes, which yields qualitative and quantitative information on proteome changes. However, to achieve this, there is need for optimum sample preparation before sample analysis, as MS results are largely dependent on sample preparation and quality [3]. The sample undergoes several steps during preparation to come up with LC–MS compatible analytes. Steps taken include protein extraction, denaturation, and digestion, which results in a peptide mixture that undergoes further peptide purification. Cell disruption and protein extraction can be achieved through physical methods, for instance sonication, which are frequently enhanced by the addition of chaotropes and detergents [4].

Sodium dodecyl sulfate (SDS), an ionic detergent, has become one of the most commonly used reagents for the solubilization of biological material [5]. In a comprehensive evaluation of 27 additives, including commonly used organic solvents, surfactants, and chaotropes, SDS demonstrates the strongest ability to solubilize membrane proteins [6]. Some studies recommend using buffer solutions with a minimum of 1% and up to 4% SDS for successful solubilization and optimal protein extraction [7,8]. Unfortunately, while coupled with the above mentioned advantages, SDS traces in the sample severely reduces enzyme activity, interferes with the performance of LC, and affects subsequent MS analysis [9]. The presence of 0.1% SDS is sufficient to reduce trypsin activity, and SDS levels above 0.01% can severely impact on chromatographical separation and suppress electrospray ionization-MS [10]. Various methods that are efficient for the removal of SDS, including affinity-based methods and electrophoretic approaches, were developed; however, they include processes that are time-consuming, and result in sample loss. As a measure to these challenges, various workflows, for instance the filter-aided sample preparation (FASP) and the single-pot, solid-phase-enhanced sample preparation (SP3), were introduced, which allow for sample processing in a single vessel [8,11]. SP3 is a novel protocol that utilizes paramagnetic beads, and provides a rapid and reliable method of proteomic sample preparation. It stands out amongst other protocols, due to its high-efficiency, speed, flexibility, and scalability [12].

Chaotropic denaturant-based in-solution digestion (ISD) is a traditional method used for protein digestion [13]. In this method, proteins are classically solubilized in urea or guanidinium hydrochloride (GnHCl), and protein cleavage is conducted through in-solution enzymatic digestion at protease-compatible concentrations of the denaturant [14]. SDS application in solution-based shotgun proteomics can be quite problematic, as the removal of SDS from samples with reversed-phase LC configuration remains challenging [15].

In this work, we aimed to decrease the usage of SDS in laboratory workflows, by finding alternate chemicals that can be used to successfully lyse and solubilize biological samples for LC–MS analysis. The efficiency of SDS in protein sample preparation was compared to that of GnHCl, a strong chaotrope and denaturing agent that does not interfere with the analysis of peptides by standard LC–MS methods [16]. SDS and GnHCl were used for the lysis and solubilization of HeLa cells and human plasma. Protein digestion was performed using the SP3 method for HeLa cells and plasma treated with both denaturants, while the ISD method was only implemented in HeLa and plasma samples lysed by GnHCl.

Indeed, the analysis of human plasma samples using LC–MS is becoming an attractive approach to investigate disease and treatment biomarkers in clinical LC–MS-based proteomics. However, the presence of highly abundant proteins (e.g., albumin) hampers the identification and quantification of less-abundant proteins of biological and clinical relevance [17]. Furthermore, in order to achieve in-depth human plasma proteome by the removal of highly abundant proteins, we coupled the use of depletion spin columns with the SP3 methodology.

Our method comparisons show the technical advantages and the proteome coverage of the SP3 approach, when using both SDS- or GnHCl-based lysis buffers, compared to the GnHCl-based ISD workflow, for the first time. The utilization of depletion spin columns before the SP3 workflow significantly increases the number of identified proteins, including those involved in neurodegenerative pathways, immune responses, and metabolism, in human plasma samples. The depletion efficacy of these commercial columns is, for the first time, described in detail.

## 2. Results

**Figure 1:**
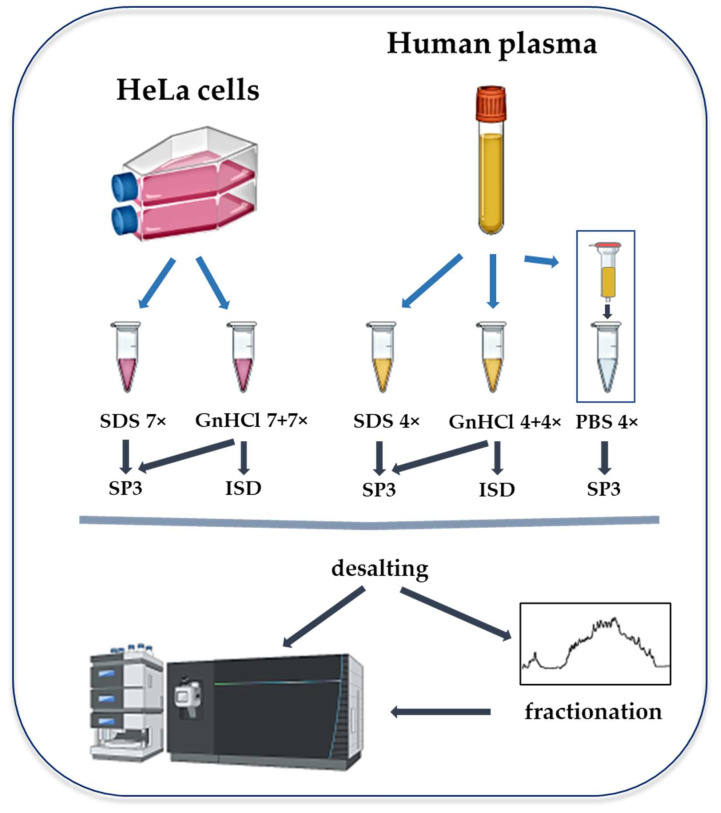
Experimental workflow of sample processing, according to the single-pot, solid-phase-enhanced sample preparation (SP3) and in-solution digestion (ISD) methodologies for MS-based proteomics analyses of HeLa cell and human plasma samples. Both cells and plasma are treated with sodium dodecyl sulfate (SDS)- or guanidinium hydrochloride (GnHCl)-based lysis buffers. Depletion mini spin columns are used with plasma samples. The depleted eluate in phosphate-buffered saline (PBS) is further processed with the SP3 workflow.

In this study, we compare the efficiency of SDS and GnHCl in the solubilization and lysis of HeLa samples, and the performance of SP3 and ISD workflows in the digestion and cleaning up of samples prior to their analysis using LC–MS. We apply these approaches to the processing of un-depleted and depleted human plasma samples (Figure 1).

### 2.1. The SP3 Protocol Outperforms the ISD Strategy with HeLa Cell Extracts

The digestion of 20 µg of protein samples from HeLa cells resuspended in an SDS- or GnHCl-based lysis buffer is carried out using the SP3 method and trypsin. The ISD method is implemented using the same amount of lysate resuspended in the GnHCl-based buffer; Lys-C and trypsin enzymes are used for protein digestion.

**Figure 2:**
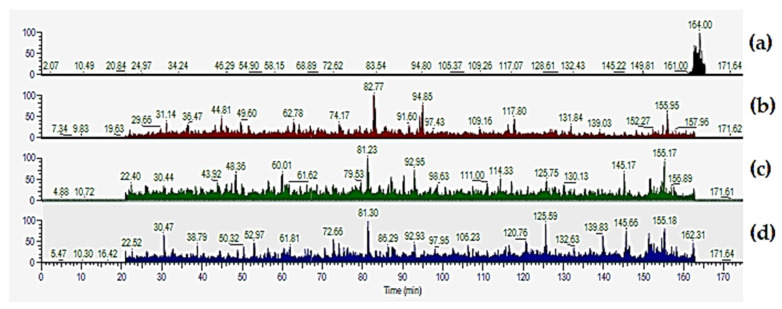
Impact of removing SDS traces in peptides solutions for LC–MS analyses. LC–MS spectra of peptides from HeLa extracts in SDS according to the standard SP3 protocol (**a**), and after introducing two extra rinses with 80% ethanol in a fresh sample tube (**b**). LC–MS spectra of HeLa peptides obtained with the SP3 and ISD methods with GnHCl buffer are shown for comparison (**c**,**d**). LC–MS run parameters are described in Section 4.6 of this manuscript.

We observe in preliminary experiments that the processing of HeLa cells extracts in SDS buffer, according to the original SP3 protocol, results in traces of SDS remaining in the peptidic solutions, which interferes with LC analysis (see Figure 2a). Following this observation, and as suggested in the original article [11], we introduce a transfer step of samples into a fresh tube, followed by two extra washes with 80% ethanol (*v*:*v*) during the sample clean-up procedure (see Materials and Methods). This results in the expected peptide chromatographic elution in the absence of SDS (Figure 2b). LC profiles of HeLa peptides obtained with the SP3/GnHCL and ISD/GnHCl workflows are shown in Figure 2c,d, respectively. To find the best performing proteomics workflow for HeLa samples, we evaluate the number of quantified proteins and peptides, the percentage of missed cleavages, and technical reproducibility.

**Figure 3:**
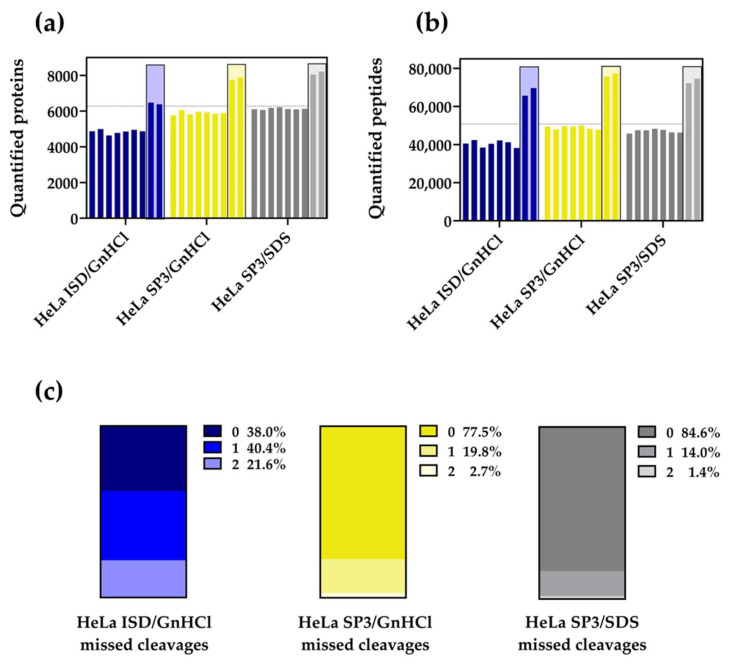
HeLa quantified proteins (**a**) and peptides (**b**) using in-solution (ISD)/GnHCl (blue), solid-pot, solid-phase-enhanced sample preparation (SP3)/GnHCl (yellow), and SP3/SDS (grey) digestion methods, from a starting protein amount of 20 µg. Framed bars represent quantification values obtained from workflows with peptide fractionation. The averaged percentage of missed cleavages (0, 1, or 2), found in all seven unfractionated replicates of each method, are shown in vertical slices plots (**c**).

With unfractionated HeLa cell samples, ISD/GnHCl quantifies 4851 ± 44 (mean ± SEM) proteins and 40,505 ± 630 (mean ± SEM) peptides (Figure 3a,b; quantification shown per replicate). SP3/GnHCl and SP3/SDS quantify 5895 ± 37 and 6131 ± 20 proteins, and 48,940 ± 345 and 47,088 ± 345 peptides, respectively. These differences are also observed when peptidic samples are fractionated; SP3/GnHCl and SP3/SDS quantify 7817 and 8136 proteins (average values), respectively.

The digestion efficiency of trypsin in the SP3 and Lys-C/trypsin in the ISD protocols using SDS and GnHCl is compared. To evaluate enzyme activity, the percentages of missed cleavages are calculated and plotted for each respective method (Figure 3c). HeLa ISD/GnHCl peptides with no missed cleavages average at 38.0%, whereas the SP3/GnHCl and SP3/SDS average at 77.5% and 84.6%, respectively. Furthermore, the percentage of peptides with one or two missed cleavages is much higher in the ISD than in the SP3 methodology, with the SP3/SDS slightly outperforming the SP3/GnHCl workflow. Taken together, our results show that the SP3-based HeLa sample processing surpasses the ISD workflows, and underline a major efficiency of protease activity and, consequently, the production of quantifiable peptides.

The quantification reproducibility of identified proteins using the different methods and lysis buffer solutions is assessed by calculating the Pearson correlation r value (Figure S1). The highest reproducibility is obtained from replicates of the same digestion method and lysis buffer solution, followed by the replicates with the same digestion method, i.e., the SP3 workflow, but different lysis buffer solutions.

### 2.2. The SP3 Protocol Enhances the Quantification of Membrane Proteomes

**Figure 4:**
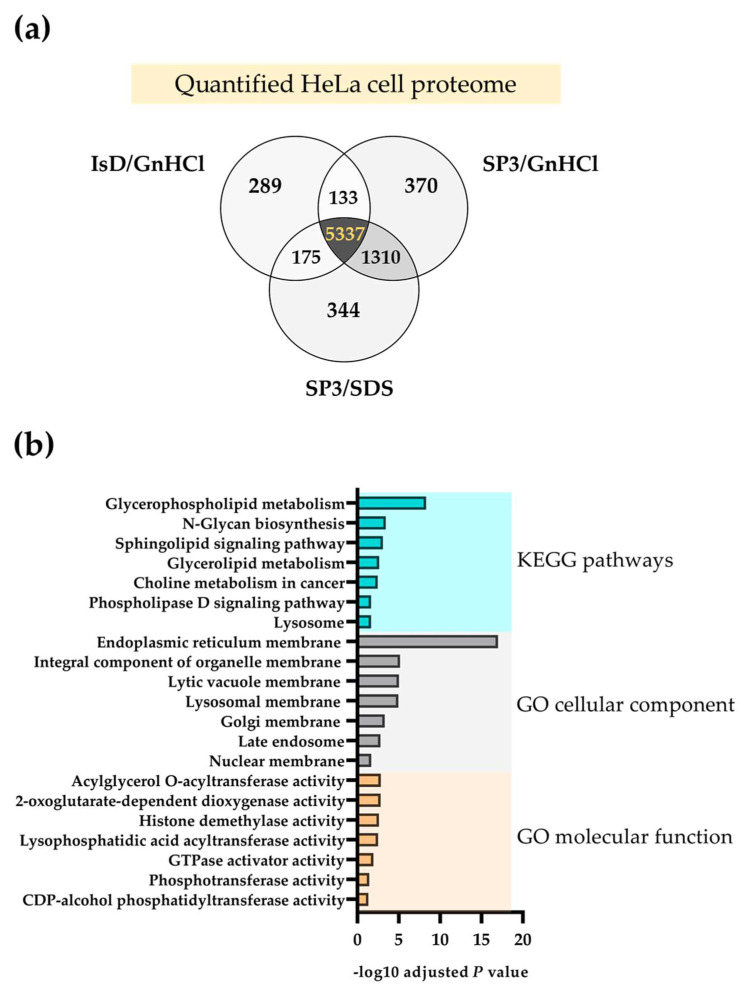
Overlap of proteins quantified in the three sample processing workflows (**a**) and characterization of the proteins exclusively quantified in the SP3 methodology (**b**). Venn diagrams are made using the gene symbols of all the HeLa cell proteins quantified in the seven unfractionated replicates. Only the dataset of 1310 proteins quantified with the SP3 approach is further submitted to KEGG pathways and gene ontology (GO) enrichment.

To further characterize the HeLa proteomes identified and quantified by the different strategies, we plot Venn diagrams with a pooled list of gene symbols observed in their corresponding unfractionated replicates. All the three methods quantify 5337 proteins, 67.1% of all the quantified proteins (Figure 4a). Approximately 17% of the quantified proteins are identified only by the SP3/GnHCl and SP3/SDS methods, and not by the ISD/GnHCl method. In order to characterize this group of 1310 proteins, gene ontology (GO) and KEGG pathway enrichments are carried out using the Enrichr webtool [18] (Figure 4b). All enriched cellular component GO terms point to intracellular membranes, whereas enriched KEGG pathways and molecular function terms comprise of proteins related to membrane-associated metabolism. These results are consistent with the broad recognition of SDS being a powerful anionic detergent that is particularly useful in membrane protein studies [5].

### 2.3. The SP3 Protocol with Depleted Plasma Samples Reaches Deeper Proteome Coverage

The ISD/GnHCl, SP3/GnHCl, and SP3/SDS workflows are also tested with human plasma samples. Quantification analyses with high-confidence proteins and peptides show that the SP3 strategy works better when compared to the ISD approach (Table 1). However, both the ISD and SP3 workflows using GnHCl as a lysis buffer quantify more peptides than the SP3/SDS protocol. As the percentage of peptides containing two missed cleavages is very similar for both the SP3/GnHCl and the SPD/SDS methods (average 2.3% vs. 1.8%), it appears that other unforeseen features lead towards a biased digestion efficiency when using the SP3 method on plasma samples with SDS-based lysis buffers. To further improve proteome coverage, we use commercial mini spin columns for the depletion of the 12 most abundant proteins in human samples, followed by the SP3 sample processing. Our results, in agreement with a recent report [19], show that a depletion step before the SP3 sample processing increases the number of quantified proteins by two-fold. Moreover, fractionation of the depleted samples further characterizes plasma proteomes, by identifying and quantifying nearly 1400 proteins.

**Figure 5:**
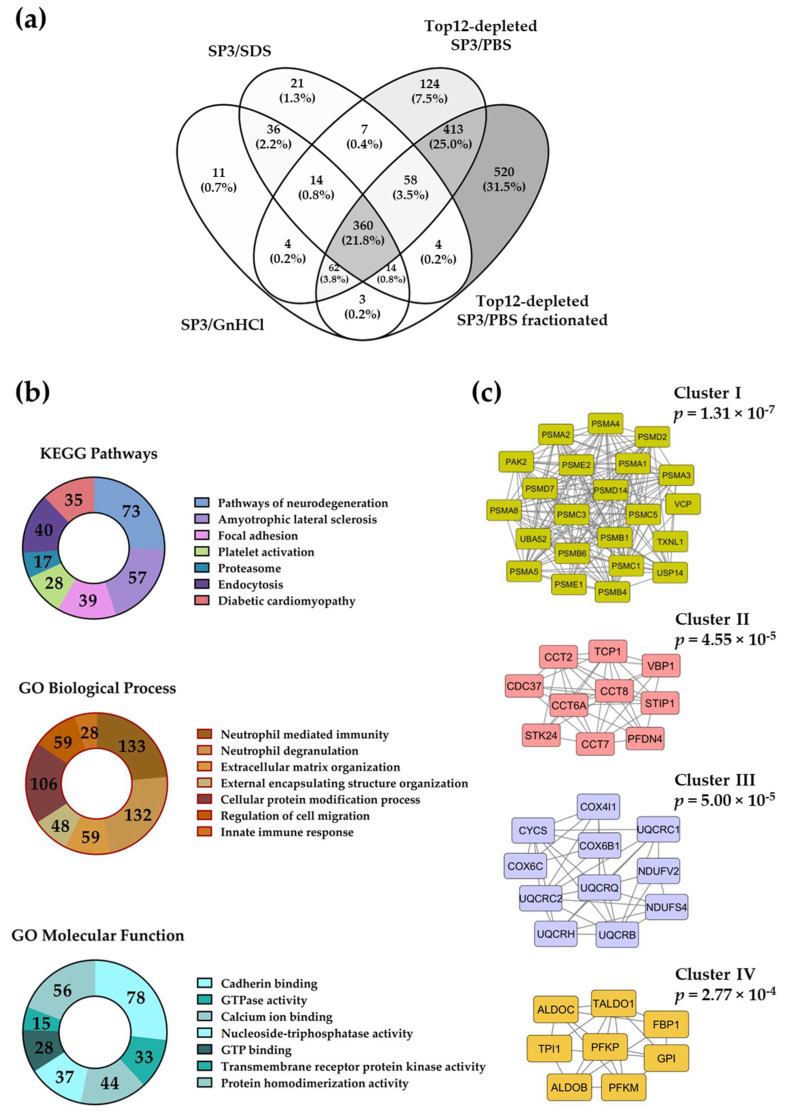
Overlap of human plasma proteins quantified in four biological replicates using SP3-based processing workflows, including those with a depletion step (**a**)**.** All the samples correspond to unfractionated datasets, except the depleted samples that are also analyzed after fractionation. A set of 933 proteins quantified only in depletion strategies is investigated for KEGG pathways and GO term enrichment. Donut plots show the number of plasma genes that overlap with the genes of the top enriched terms (**b**). Most significant protein–protein interactions (PPI) networks from the 933 proteins dataset found in STRING database, visualized and analyzed with Cytoscape and ClusterONE, respectively (**c**). Four clusters with the highest significance of cohesiveness are shown with *p* values of a one-sided Mann–Whitney U test.

In order to describe the plasma proteome that becomes identifiable and quantifiable after the depletion step, we overlap protein datasets quantified in the different depleted and un-depleted SP3 workflows (Figure 5a). KEGG pathways and GO analyses show that neurodegeneration pathways, as well as neutrophil-mediated immune responses, protein modification, cadherin/GTP/calcium ion binding, and GTPase activity GO terms, are significantly enriched in a dataset comprised of 933 proteins quantified after depletion in fractionated and non-fractionated samples (Figure 5b).

A protein–protein interaction (PPI) network study of the 933 proteins dataset reveals a large cluster, containing mostly proteasomal subunits (Figure 5c, cluster I). Three other significant clusters (cluster II–IV) contain subunits of the chaperonin CCT, the proteins in charge of the mitochondrial electron transport, and fructose 1,6-bisphophate aldolases.

### 2.4. Evaluation of the Removal of Highly Abundant Proteins in Plasma Samples with Commercial Mini Spin Depletion Columns

In order to examine the performance of the mini spin depletion columns, we examine the number of peptide–spectrum matches (PSM) detected for each of the 12 abundant proteins after SP3-based sample processing, with and without the depletion step, and after the ISD protocol. Although the numbers of PSMs of these 12 proteins varied among the different workflows that did not employ the depletion step, we observe that, when compared to the expected 95% removal, the depletion mini spin columns substantially decrease the abundance of albumin, transferrin, IgA, and IgG (Figure S2). Alpha-2-macroglobulin, haptoglobin, and IgM abundances decrease, to a lesser extent. However, we do not detect any removing effect of alpha-1-acid glycoprotein, alpha-1-antitrypsin, apolipoprotein AI and AII, or fibrinogen.

Taken together, even though the mini spin depletion columns might not efficiently remove all 12 highly abundant proteins in plasma samples, decreased levels of highly abundant plasma proteins, such as albumin, transferrin, and immunoglobulins, appears sufficient to have a positive effect in the quantification of a higher number of plasma proteins.

## 3. Discussion

Sample processing is a determinant step in LC–MS proteomics workflows. In this manuscript, we show that the SP3 method performs better than the ISD method.

The SP3 protocol, with either an SDS- or GnHCl-based lysis buffer, performs relatively similarly, which highlights that the combination of any of these buffers and the SP3 beads are effective methods for LC–MS analysis of HeLa cell samples. Although a double digestion with Lys-C and trypsin enzymes is carried out in the ISD/GnHCl approach, sample dilution to reduce the amount of guanidinium before overnight digestion might negatively affect protease performance. Nonetheless, bead-free workflows are still highly used in proteomics laboratories, especially in the processing of the large amounts of lysate required for post-translation modification (PTM) studies [20,21]. Alternatively, although the SP3 method was widely used in the preparation of small amounts of proteomics samples, it was recently adapted for the processing of up to 10 mg of proteins [22].

Plasma is a more complex type of sample, which is highly rich in proteins [16]. It is well-known that plasma proteomics face the challenge of deep protein identification, as plasma samples contain highly abundant proteins, such as albumin and fibrinogen, that hinder the analysis of the global plasma proteome [23]. Our experiments show that processing raw human plasma samples with the ISD/GnHCl, SP3/GnHCl, and SP3/SDS protocols perform similarly. In order to achieve a higher plasma proteome coverage, an extra depletion step and peptide fractionation are widely used strategies. Examples of depletion strategies involve multiple-use, high-performance liquid chromatography (HPLC) columns, for depletion of up to 14 proteins [24,25], IgY ultra-high depletion columns [23], multiple-use mini spin columns, and, recently, single-use depletion mini spin columns [19]. The latter approach was recently investigated in detail, reporting the identification of approximately 2000 human plasma proteins, and excellent quantification reproducibility using a Top14 abundant protein depletion mini spin column (Thermo Fisher Scientific, Waltham, MA, USA) [19]. The results of our study also support the use of depletion mini spin columns, increasing by 2-fold and by 3.5-fold the number of proteins quantified in unfractionated and fractionated plasma samples, respectively. Although the PSM analyses of the 12 most abundant proteins in depleted and un-depleted samples show that the performance of these mini spin columns is not optimal, the partial removal of albumin, immunoglobulins, haptoglobin, and transferrin appears to enhance the proteome coverage of depleted samples. Nevertheless, our depleted plasma datasets do not succeed in identifying lower-abundant proteins, such as gut hormones and gut integrity markers, indicating how challenging the high dynamic range of plasma protein expression remains.

The use of Top14 depletion midi spin columns with a load of 40 µL of plasma, followed by high-resolution isoelectric focusing (HiRIEF) peptide fractionation, does not show a substantial increase in the number of quantified proteins [19]. However, the performance of these columns with the maximum sample load of 100 µL, followed by peptide fractionation approaches such as high pH reversed-phase chromatography remains to be investigated.

Besides depletion spin columns, new approaches were introduced to deeply study plasma samples in clinical proteomics. One of these involves the use of engineered nanoparticle protein coronas, which allows the detection of more than 2000 plasma proteins [26]. This, and future initiatives, might overcome the sensitivity and depth challenges that we currently face in clinical proteomics studies with plasma samples.

## 4. Materials and Methods

### 4.1. Cell Culture and Plasma

HeLa cells were cultured at the Department of Biomedicine of the University of Bergen, and kindly provided by Dr. Stacey Dmello. Human plasma samples were provided by Dr. Ingeborg Brønstad and Prof. Kjell-Morten Myhr at the Department of Clinical Medicine of the University of Bergen. EDTA whole blood was centrifuged at 1800× *g* at 4 °C for 10 min. Plasma samples were stored in aliquots at −80 °C until analysis. Ethical approval and informed consent to study human plasma samples are described in the institutional review board and informed consent statements at the end of this manuscript.

### 4.2. Protein Extraction

#### 4.2.1. GnHCl- and SDS-Based Lysis

HeLa cells were resuspended in a GnHCl lysis (6 M GnHCl and 0.1 M Tris-HCl pH 8.5) or SDS lysis buffer (4% SDS and 0.1 M Tris-HCl pH 8.5), containing 5 mM tris(2-carboxyethyl)phosphine (TCEP) and 10 mM chloroacetamide (CAA). Protein concentration was determined using the Pierce Coomassie Plus (Bradford) Protein Assay or the Pierce BCA Protein Assay kit (Thermo Fisher Scientific, Waltham, MA, USA) [21]. Twenty µg of protein was aliquoted, and topped up with either GnHCl lysis or SDS lysis buffer, to a final volume of 50 µL. One hundred micrograms of plasma proteins, determined by the Pierce BCA Protein Assay kit, were dissolved in a final volume of 100 µL of the GnHCl or SDS buffer. All samples were boiled at 95 °C for 10 min, and microtip sonicated.

#### 4.2.2. Depletion of Abundant Plasma Proteins

The Pierce Top12 abundant protein (α1-acid glycoprotein, α1-antitrypsin, α2-macroglobulin, albumin, apolipoprotein AI, apolipoprotein AII, fibrinogen, haptoglobin, IgA, IgG, IgM, and transferrin) depletion spin columns (Thermo Fisher Scientific) were used with 10 µL of human plasma. A total of 5 mM TCEP and 10 mM CAA were added to 450 µL of depleted samples containing 70 µg of protein on average. Samples were boiled at 95 °C for 10 min.

### 4.3. Protein Sample Digestion

Seven technical replicates of HeLa cell extracts and four different plasma samples (as biological replicates) were prepared for every workflow to be tested.

#### 4.3.1. ISD Protein Digestion

ISD digestion was performed on both GnHCl-treated HeLa cells and un-depleted plasma samples, as previously described, with minor modifications [21]. Lys-C (FUJIFILM Wako Pure Chemical Corporation, Osaka, Japan) was added to the samples in an enzyme/protein ratio of 1:100 (*w*/*w*), and incubated for 1 h at 37 °C. Samples were then diluted 6-fold with 50 mM Tris-HCl pH 8.5, and digested overnight at 37 °C using trypsin (Promega, Madison, WI, USA) at an enzyme/protein ratio of 1:50 (*w*/*w*).

#### 4.3.2. Protein Digestion with the SP3 Methodology

HeLa cells and un-depleted plasma samples lysed with the GnHCl and SDS buffers were all digested using the SP3 method [11]. GnHCl-lysed samples were diluted to a GnHCl concentration of 3 M, since concentrations above that can be incompatible with the SP3 approach. Digestion was performed using a 1:1 ratio of magnetic carboxylate-modified beads (GE Healthcare, Chicago, IL, USA). A bead solution at 100 µg/µL was added to each replicate to a bead:protein ratio of 10:1 (*w*/*w*). Proteins solubilized in SDS were additionally washed twice with the 80% (*v*:*v*) ethanol solution on a fresh tube, in order to remove possible traces of SDS. Depleted plasma samples in PBS were processed with the standard workflow of the SP3 method.

### 4.4. Peptide Cleaning

SP3- and ISD-processed samples were acidified to a pH of 2–2.5. Desalting was performed using the Oasis HLB 96-well µElution Plate, 30 mm (Waters, Milford, MA, USA), according to the manufacturer’s instructions. Dried peptides were resuspended in a solution containing 0.5% (*v*:*v*) formic acid (FA) and 2% (*v*:*v*) ACN. Peptide concentration of all the samples was determined using the Nanodrop One Microvolume UV-Vis spectrophotometer (Thermo Fisher Scientific). Samples were then stored at −20 °C, until LC–MS analysis.

### 4.5. Offline High pH Reversed-Phase HPLC Fractionation

For deep proteome analysis of HeLa cell and plasma samples, 20 µg of peptides was resuspended in 10 mM ammonium formate pH 7.9 (buffer A), and fractionated using a reversed-phase XSelect CSH C18 3.5 µm 1 × 150 mm column (Waters), on a 1260 Infinity LC system using ChemStation software (Agilent, Santa Clara, CA, USA). The system operated at 50 µL/min. Peptides were separated by a multi-step gradient as follows: 0–1.5 min, 5% of 90% ACN (buffer B); 1.5–2 min, 5%–10% buffer B; 2–25 min, 10%–55% buffer B; 25–28 min, 55%–95% buffer B; 28–33 min 95% buffer B; 33–35 min 95%–5% buffer B; 35–45 min, 5% buffer B.

### 4.6. LC-MS/MS Analysis

Unfractionated samples containing 0.75–0.80 µg tryptic peptides were injected into an Ultimate 3000 RSLC system (Thermo Fisher Scientific), which was online and coupled to the Orbitrap Eclipse Tribrid mass spectrometer that consisted of an EASY-IC/ETD/PTCR ion source, together with a FAIMS Pro interface (Thermo Fisher Scientific, San Jose, CA, USA). Firstly, the samples were loaded and desalted on a pre-column (Acclaim PepMap 100, 2 cm × 75 µm ID nanoViper column, packed with 3 µm C18 beads), at a flow rate of 6 µL/min with 0.1% (*v*:*v*) trifluoroacetic acid (TFA). Peptides were then separated during a biphasic ACN gradient with 0.1% FA as buffer and 0.1% FA in ACN as buffer B, on a 50 cm analytical column (Acclaim PepMap 100, 50 cm × 75 µm ID nanoViper column, packed with 2 µm C18 beads) at a flow rate of 200 nL/min. The gradient composition over 180 min was as follows: trapping over 5 min with 5% B, followed by 5–7% B for 1 min, 7–22% B for the next 129 min, 22–28% for 14 min, and 28–80% B for 7 min, hold at 80% B for 18 min, and then ramp to 5% B for 6 min. In order to elute sticky plasma peptides from the system, a similar gradient profile, reaching 95% B, was applied. The trap and the valve were subjected to a 15 min wash run with isopropanol after each sample run, to minimize chances of sample carryover effect.

Tune v3.5.3881.15 and Xcalibur 4.5 software were used for instrument control. The MS1 resolution was 120,000 and the scan range 375–1500 m/z; AGC was set to standard, maximum injection time was automatic, and the RF lens was set at 30%. The intensity threshold was set at 5.0 × 10^4^ and the dynamic exclusion lasted for 30 s. The MS2 scans consisted of HCD with collision energy at 30%, quadrupole isolation window at 4 m/z, and Orbitrap resolution of 15,000. The AGC was set to standard, and maximum injection time was set at 75 ms. For FAIMS, the standard resolution mode was used, with a total gas flowrate of 3.8 L/min. CVs were set to −45, −65, and −80. As a quality control for the LC–MS/MS system, 100 ng of a HeLa digest was run at the beginning, during, and at the end of the sample sequence (data not shown).

Fractionated HeLa cells and plasma cells were run using the same amount of peptides and LC–MS/MS parameters as described, with the exception of the use of a shorter gradient of 120 min.

Raw files are available, as described in the data availability statement at the end of this manuscript.

### 4.7. Statistics and Data Processing

Sequest HT database search engine [27], with Percolator validation [28] (FDR < 0.01), was used for searching the raw LC–MS/MS files in Proteome Discoverer software v2.5 (Thermo Fisher Scientific). The search was conducted against the reviewed Swiss-Prot human database that was downloaded (as a fasta file containing canonical and isoforms) on 30 April 2021. The default settings were applied, and normalization was performed using the sum of all peptide amounts, while protein abundances were calculated by summing sample abundances of the connected peptide groups. The software performed protein grouping, and reported results were filtered for master proteins (master proteins are referred to as proteins in this manuscript for simplicity purposes). Following this, Perseus software v1.6.15.0 was used for further processing of the imported normalized protein abundances [29,30]. Briefly, the data was filtered to remove contaminants, and proteins with low and medium FDR confidence scores. Following this, log2 data conversion was completed, and the median was subtracted from all values. Scatterplots showing the Pearson correlation among replicates and workflows were created in the Perseus platform. Column plots for the number of quantified proteins and peptides, and boxplots for the missed cleavage data were created using GraphPad Prism 9.0.0 (GraphPad Software, San Diego, CA, USA). The Venny online tool was utilized in the creation of Venn diagrams and generation of overlapped protein data, which was further used to create GO and KEGG pathway enrichment plots using Enrichr [18]. GraphPad Prism 9.0.0 was used in plotting the enrichment analyses. PPI networks were obtained using the STRING database version 11.5, with interactions derived from experiments and databases at a high-confidence score of 0.9 [31]. Networks were visualized using the Cytoscape platform version 3.7.2 [32]. The ClusterONE plugin was used to identify protein groups of high cohesiveness [33]. The workflow chart of our experimental strategy was created using BioRender (BioRender.com; accessed on the 10 December 2021).

## 5. Conclusions

In the present study, we tested three methods, ISD/GnHCl, SP3/GnHCl, and SP3/SDS with HeLa cells and human plasma samples. The SP3/SDS and SP3/GnHCl methods outperform the ISD/GnHCl in the quantification of proteins and peptides of HeLa cells, whereas the three methods produce similar results in protein and peptide quantification of the plasma samples. Analysis of the enrichment data of the quantified HeLa proteins reveals that SP3 methods are more efficient than the ISD/GnHCl protocol in quantifying membrane proteins. The SP3 workflow applied to depleted plasma samples, followed by high pH reversed-phase chromatography, quantified ~1000 more proteins than un-depleted workflows.

## Figures and Tables

**Table 1:** High-confidence quantified proteins and peptides in human plasma samples using ISD- and SP3-based workflows.

	ISD/GnHCl	SP3/GnHCl	SP3/SDS	Top12-Depleted SP3/PBS	Top12-Depleted SP3/PBS & Fractionated
Quantifiedproteins [1]	361 (35)	397 (32)	411 (35)	833 (76)	1397 (48) [2]
Quantifiedpeptides [1]	3822 (309)	3726 (172)	3117 (134)	5860 (624)	8431 (617) [2]

[1] Data are expressed as mean of four biological replicates (SD). [2] Data are expressed as mean of two biological replicates that underwent fractionation (SD).

## Data Availability

The data presented in this study are openly available in the ProteomeXchange consortium via the PRIDE partner repository, with dataset identifier PXD031918 for the HeLa samples and PXD031919 for the human plasma samples.

## Supplementary Materials

The following supporting information can be downloaded at: https://www.mdpi.com/article/10.3390/molecules27113390/s1, Figure S1: Pearson correlation plots of protein abundance; Figure S2: Effect of the use of depletion mini-spin columns on removal of most abundant proteins in human plasma samples.
