# Supplementary material for: Comparing Efficiency of Lysis Buffer Solutions and Sample Preparation Methods for Liquid Chromatography–Mass Spectrometry Analysis of Human Cells and Plasma
Source: Molecules. 2022 May 25;27(11):3390. doi: 10.3390/molecules27113390 (PMC9181984; doi:10.3390/molecules27113390)
Supplement: Supplementary file 1 [file molecules-27-03390-s001.zip › molecules-1653549-supplementary.pdf]

Supplementary materials

Comparing efficiency of lysis buffer solutions and sample preparation methods for liquid chromatography-mass spectrometry analysis of human cells and plasma

Lasse Neset [1,†], Gracious Takayidza [1,†], Frode S. Berven [1] and Maria Hernandez-Valladares [1, 2, 3,*,†]

[1] The Department of Biomedicine, University of Bergen, Jonas Lies vei 91, 5009 Bergen, Norway; Lasse.Neset@student.uib.no; Gracious.Takayidza@student.uib.no; Frode. Berven@uib.no
[2] Department of Clinical Science, University of Bergen, Jonas Lies vei 87, 5021 Bergen, Norway; Maria.Hernandez-Valladares@uib.no
[3] Department of Physical Chemistry, University of Granada, Campus Fuentenueva s/n, 18071 Granada, Spain
* Correspondence: Maria.Hernandez-Valladares@uib.no; Tel.: +47 55586368
† These authors contributed equally to this work

Supplementary material

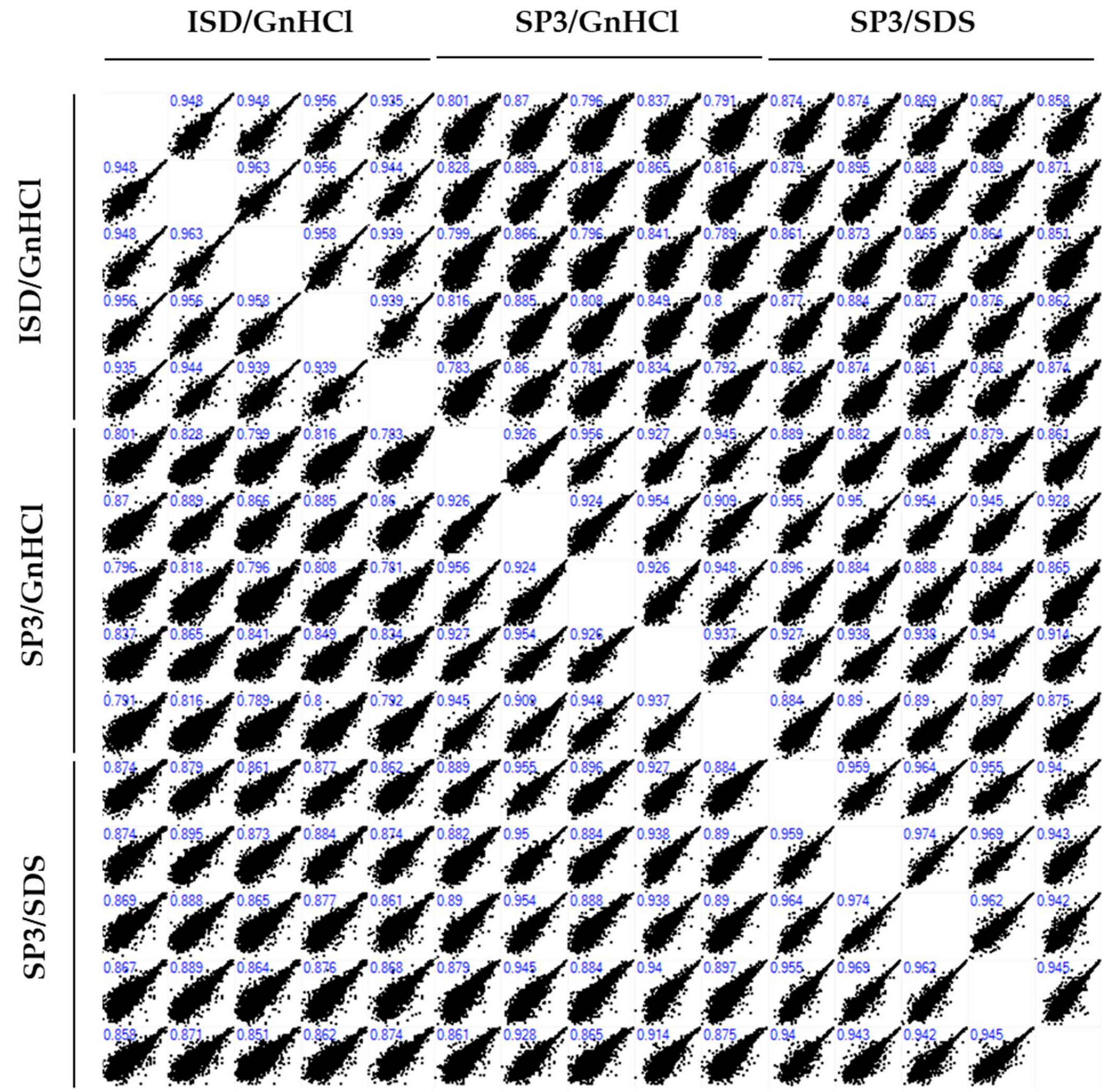

Figure S1. Pearson correlation plots of protein abundance. Quantifications are shown for five out of seven unfractionated replicates from each workflow. Pearson correlation *r* values are shown in blue. The plots are done in Perseus software [1,2].

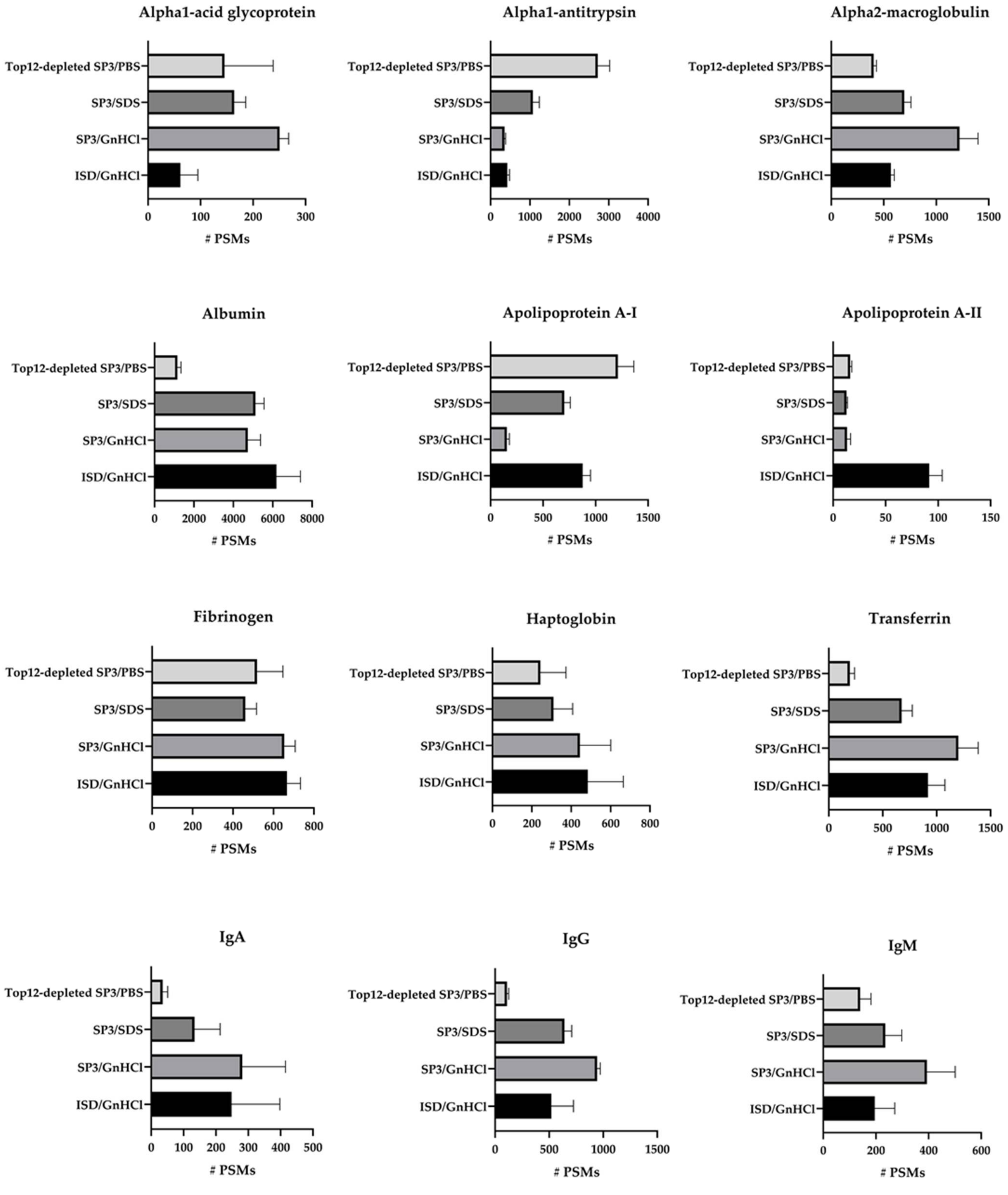

Figure S2. Effect of the use of depletion mini spin columns on the removal of most abundant proteins in human plasma samples. Bar plots show the number of peptide spectrum matches (PSMs) for each

of the twelve highly abundant proteins identified in each workflow carried out without further fractionation. The bars expressed average values with SD of four biological replicates.

References

1. Tyanova, S.; Cox, J., Perseus: A Bioinformatics Platform for Integrative Analysis of Proteomics Data in Cancer Research. *Methods Mol. Biol.* **2018,** *1711*, 133–148.
2. Tyanova, S.; Temu, T.; Sinitcyn, P.; Carlson, A.; Hein, M. Y.; Geiger, T.; Mann, M.; Cox, J., The Perseus computational platform for comprehensive analysis of (prote)omics data. *Nat. Methods* **2016,** *13*, 731–740.
